# Reconstructing the gradient source position from steady-state fluxes to small receptors

**DOI:** 10.1038/s41598-018-19355-5

**Published:** 2018-01-17

**Authors:** Ulrich Dobramysl, David Holcman

**Affiliations:** 1 Cancer Research UK Gurdon Institute, University of Cambridge, Cambridge, United Kingdom; 2 Ecole Normale Supérieure 46 rue d’Ulm 75005, Paris, France; 3 DAMPT, University of Cambrdige, Storeys way, Cambridge, CB30DS United Kingdom

## Abstract

Recovering the position of a source from the fluxes of diffusing particles through small receptors allows a biological cell to determine its relative position, spatial localization and guide it to a final target. However, how a source can be recovered from point fluxes remains unclear. Using the Narrow Escape approach for an open domain, we compute the diffusion fluxes of Brownian particles generated by a steady-state gradient from a single source through small holes distributed on a surface in two dimensions. We find that the location of a source can be recovered when there are at least 3 receptors and the source is positioned no further than 10 cell radii away, but this condition is not necessary in a narrow strip. The present approach provides a computational basis for the first step of direction sensing of a gradient at a single cell level.

## Introduction

Sensing a molecular gradient made of cue concentration is the first step to transform cell positional information into a genetic specialization and a differentiation signal^1^. During axonal growth and guidance, the growth cone (the tip of a neuron) uses the concentration of morphogens^2,3^ to decide whether or not to continue moving, stop, turn right or left. Bacteria and spermatozoa in particular are able to orient themselves in a chemotaxis gradient^4–10^. However, how a cell senses an external gradient concentration depends on its ability to estimate the fluxes of cues. These fluxes have been computed assuming that cues are fully or partially absorbed uniformly at the surface of a detecting ball^11^. These computations are used to estimate the sensitivity of the local concentration. This, however, is insufficient to establish the orientation of the source of the gradient. Our aim is to clarify how a cell, which is only a few microns in size, can detect the direction of a source. In particular, our main focus is on the directional sensing of cues in neuronal growth cones, although the presented techniques should also be applicable to a wider range of cells.

The first step of differentiating left from right certainly has to involve the spatial difference in the binding flux of external cues. A receptor can use two mode of decoding: it can respond to the rate or to the binding time^5^. We are exploring here the first possibility, that corresponds to a diffusion-limited regime where any cue hitting a receptor will immediately lead to receptor activation. Examples are chemoattractants that bind with high affinity to the receptor guanylyl cyclase in sperm in order to monitor the rate of binding events rather than the receptor occupancy. Other examples are odorants and endocrine disrupting chemicals that activate channels with fast activation. In neurons TRPV1 channel integrates various stimuli. In addition, we do not consider here a possible time integration of the external cues signal, as described for sperms, which is based on the rate of changes in calcium concentration directly modulated by an external gradient^12^. We are mainly concerned with growth cone navigation in the developing brain, for which there is no obvious time integration, because the motion of a growth cone is much slower than the binding time of cues^13^ (see also^14^).

Our model for cell direction detection uses a reflecting disk covered with small receptors. The receptors are perfect absorbers for Brownian particles (cues), emanating from a point source. Computing the fluxes of Brownian particles to small targets is part of the Narrow Escape Theory^15–17^, but this theory cannot be applied directly to open and unbounded domains, because the mean passage time for particles to hit any small target is infinite. To avoid this difficulty, we neglect the receptor binding time of diffusing molecules and consider that cell sensing is possible via direct measurement of the diffusion flux. However, we do not account for any further cellular transduction cascade that translates receptor local activation into an internal signal. A receptor-local memory mechanism is necessary in order to prevent loss of directional information on the gradient due to homogenization of the downstream transduced signal (concentration of a second messenger or surface molecules) inside the cell. Therefore, asymmetric fluxes at the receptor level should lead to an asymmetrical transduction inside the cell. Hence, we do not replace receptors by a homogenized boundary condition that would render measurements of spatial flux differences impossible.

In this letter, we first compute the fluxes of diffusing molecules to small targets (*N* = 2 and 3) located on the surface of a detecting disk. We evaluate the effect of different receptor arrangements and also study the influence of an infinitely long, confining narrow strip. Secondly, we estimate the maximum distance from a source at which a given concentration can be detected with a pre-defined accuracy. Finally, we study how the location of a source can be recovered from the difference of fluxes. We demonstrate that the source position of a gradient can be reconstructed or triangulated with three receptors, while sensing of the concentration level can be achieved with two only.

## Results

### Diffusion fluxes through narrow windows

The probability density function *p*_*t*_(***x***, ***x***_0_) for a Brownian particle generated at location ***x***_0_ that can be absorbed on the boundary of a detecting two-dimensional disk or radius *R*, Ω = *D*(*R*), by windows $\partial {{\rm{\Omega }}}_{1}\cup \ldots \cup \partial {{\rm{\Omega }}}_{N}$ located on the surface ∂Ω of said disk Ω satisfies

1 $$\frac{\partial {p}_{t}}{\partial t}=D{\rm{\Delta }}{p}_{t}$$

2 $$\begin{array}{rcl}{p}_{t}({\boldsymbol{x}},{{\boldsymbol{x}}}_{0}) & = & \delta ({\boldsymbol{x}}-{{\boldsymbol{x}}}_{0})\,{\rm{for}}\,{\boldsymbol{x}}\in {{\mathbb{R}}}^{2}-{\rm{\Omega }}\,{\rm{and}}\,t=0\\ \frac{\partial {p}_{t}}{\partial {\boldsymbol{n}}}({\boldsymbol{x}},{{\boldsymbol{x}}}_{0}) & = & 0\,{\rm{for}}\,{\boldsymbol{x}}\in \partial {\rm{\Omega }}-(\partial {{\rm{\Omega }}}_{1}\cup \ldots \cup \partial {{\rm{\Omega }}}_{N})\\ {p}_{t}({\boldsymbol{x}},{{\boldsymbol{x}}}_{0}) & = & 0\,{\rm{for}}\,{\boldsymbol{x}}\in \partial {{\rm{\Omega }}}_{1}\cup \ldots \cup \partial {{\rm{\Omega }}}_{N}\mathrm{.}\end{array}$$

The reflecting boundary condition at $\partial {\rm{\Omega }}-(\partial {{\rm{\Omega }}}_{1}\cup \ldots \cup \partial {{\rm{\Omega }}}_{N})$ accounts for the impenetrable boundary so that diffusing molecules are reflected on the surface. The absorbing boundary condition on each of the windows $\partial {{\rm{\Omega }}}_{1}\cup \ldots \cup \partial {{\rm{\Omega }}}_{N}$ represents rapid binding with a diffusion limited activation rate. The window sizes are identical and equal to $|\partial {{\rm{\Omega }}}_{1}|=\varepsilon \ll 1$. The steady-state probability density *P*_0_(***x***) is computed by solving the mixed boundary value problem for the Laplace equation^17^

3 $$\begin{array}{rcl}-D{\rm{\Delta }}{P}_{0}({\boldsymbol{x}}) & = & \delta ({\boldsymbol{x}}-{{\boldsymbol{x}}}_{0})\,{\rm{for}}\,{\boldsymbol{x}}\in {{\mathbb{R}}}^{2}-{\rm{\Omega }}\\ \frac{\partial {P}_{0}}{\partial {\boldsymbol{n}}}({\boldsymbol{x}}) & = & 0\,{\rm{for}}\,{\boldsymbol{x}}\in \partial {\rm{\Omega }}-(\partial {{\rm{\Omega }}}_{1}\cup \ldots \cup \partial {{\rm{\Omega }}}_{N})\\ {P}_{0}({\boldsymbol{x}}) & = & 0\,{\rm{for}}\,{\boldsymbol{x}}\in \partial {{\rm{\Omega }}}_{1}\cup \ldots \cup \partial {{\rm{\Omega }}}_{N}\mathrm{.}\end{array}$$

Although the density *P*_0_(***x***) is non-normalizable in two dimensions, we are only interested in the splitting probability between windows, i.e. the normalized steady-state flux at window *k*,

4 $${P}_{k}=\frac{{\int }_{\partial {{\rm{\Omega }}}_{k}}\,\frac{\partial {P}_{0}({\boldsymbol{x}})}{\partial {\boldsymbol{n}}}d{S}_{{\boldsymbol{x}}}}{{\sum }_{q}{\int }_{\partial {{\rm{\Omega }}}_{q}}\,\frac{\partial {P}_{0}({\boldsymbol{x}})}{\partial {\boldsymbol{n}}}d{S}_{{\boldsymbol{x}}}}.$$

Due to the recurrent property of the Brownian motion in two dimensions, the probability to hit a window before going to infinity is one, thus

5 $$\sum _{q}\,{\int }_{\partial {{\rm{\Omega }}}_{q}}\,\frac{\partial {P}_{0}({\boldsymbol{x}})}{\partial {\boldsymbol{n}}}d{S}_{{\boldsymbol{x}}}=1.$$

The fluxes for *N* = 2 windows can be computed using the Green’s function *G*(***x***, ***y***) of the domain using matched asymptotic expansion^18,19^ and involves a Green’s function Matrix in general. However, using identity Eq. (5), it is sufficient to compute only one probability and we get

6 $${P}_{2}=\frac{1}{2}+\frac{\pi }{2}\frac{G({{\boldsymbol{x}}}_{1},{{\boldsymbol{x}}}_{0})-G({{\boldsymbol{x}}}_{2},{{\boldsymbol{x}}}_{0})}{\{\,\mathrm{log}\,|{{\boldsymbol{x}}}_{1}-{{\boldsymbol{x}}}_{2}|-\,\mathrm{log}\,\varepsilon \}},$$

where the external Neumann-Green’s function of a disk Ω = *D*(*R*) of radius *R*, for ${\boldsymbol{x}},{\boldsymbol{y}}\in {{\mathbb{R}}}^{2}-B(R)$ is

7 $$G({\boldsymbol{x}},{\boldsymbol{y}})=\frac{-1}{2\pi }(\mathrm{ln}\,|{\boldsymbol{x}}-{\boldsymbol{y}}|+\,\mathrm{ln}|\frac{{R}^{2}}{|{\boldsymbol{x}}{|}^{2}}{\boldsymbol{x}}-{\boldsymbol{y}}|).$$

**Figure 1:**
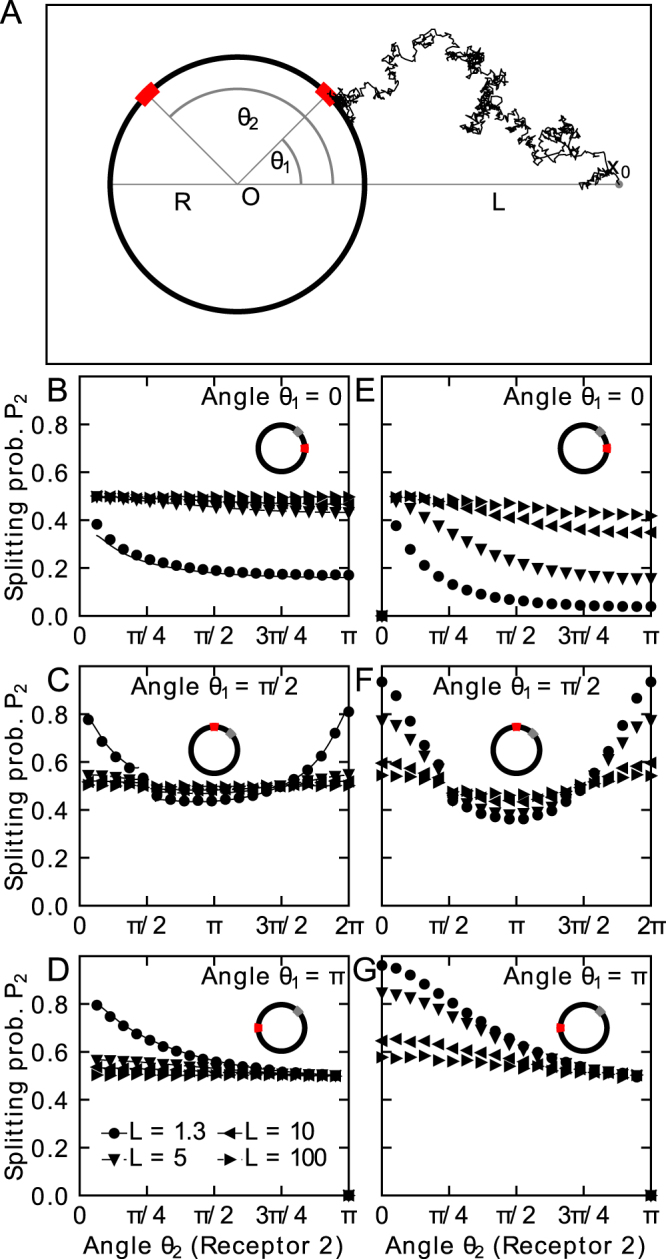
Diffusion fluxes to small windows on the disk surface. (**A**) Schematic representation of a mixed stochastic simulation of Brownian particles released at position *x*_0_ at a distance *L* = |*x*_0_| from the origin *O*. Two windows of size $2\epsilon$ are placed on the circumference of the disk of radius *R* in two dimensions or the equator of a sphere in three dimensions at angles *θ*_1_ and *θ*_2_ with the *x*-axis. Brownian particles are injected at a distance *R*_*e*_. (**B**) Splitting probability (normalized flux) at window 2 in two dimensions with angle *θ*_1_ = 0, (**C**) *θ*_1_ = *π*/2 (the jump in the analytical solution at *π*/2 emerges due to divergence when windows overlap), and (**D**) *θ*_1_ = *π*. Simulations (markers) are compared to analytical solutions (solid lines). (**E**) Splitting probability at window 2 in three dimensions (flux normalized to the total flux absorbed by any of the two windows) with angle *θ*_1_ = 0, (**F**) *θ*_1_ = *π*/2, and (**G**) *θ*_1_ = *π*.

To evaluate how the probability *P*_2_ changes with the distance of the source ***x***_0_ and the relative position of the windows, we compare Brownian simulations with the analytical expression (6) for a disk (Fig. 1A). For the simulations, we generated Brownian trajectories near the disk (on the surface of a disk of radius *R*_*e*_) according to the exit point distribution of a process from an internal disk^20^. Interestingly, already at a distance of *L* = 10*R*, the absolute difference between the fluxes Δ*P* = |*P*_1_ − *P*_2_| is within 5%, making it impossible to determine source direction or concentration differences in a noisy environment (as long as the amplitude is below the threshold of 5%). This result is independent of the window positions and Δ*P* → 0 as *L* increases, see Fig. 1B–D.

### Diffusion in a narrow strip

**Figure 2:**
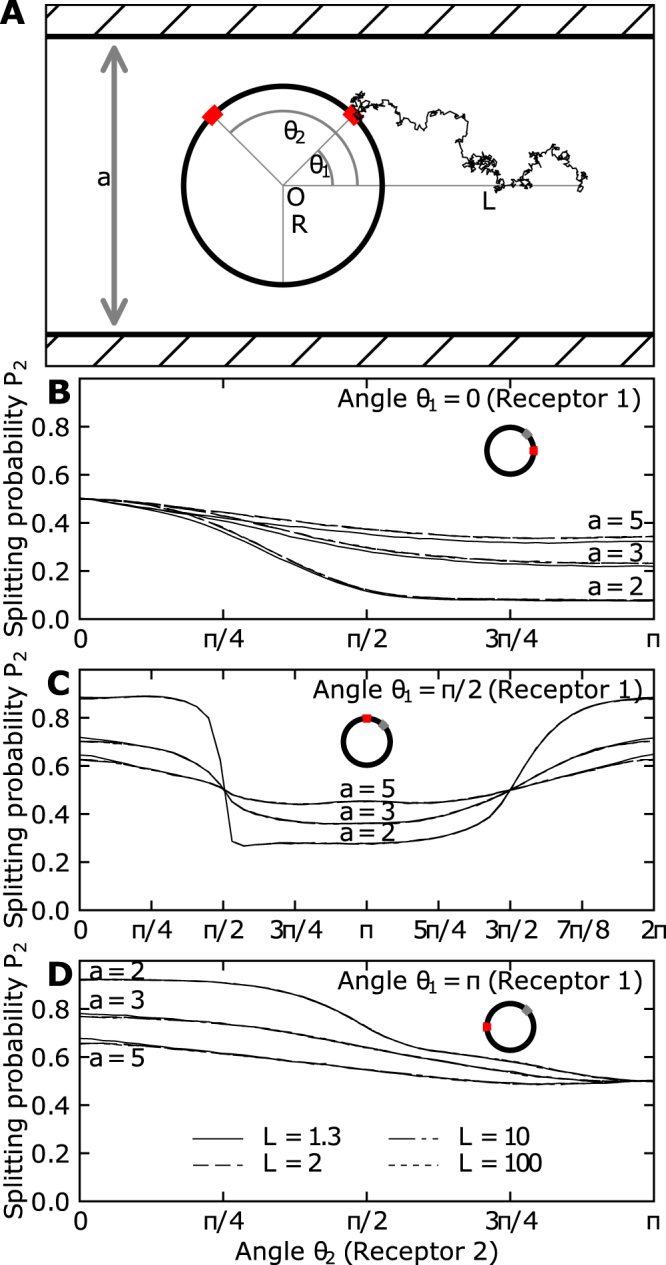
Diffusion fluxes to small windows for a disk in a narrow strip of width *a*. (**A**) Scheme of the mixed stochastic simulations of Brownian particles confined in the strip and released at position *x*_0_ at a distance *L* = |*x*_0_| from the origin *O*. Two windows of size $2\epsilon$ are placed on the circumference of the disk of radius *R* at angles *θ*_1_ and *θ*_2_ with the *x*-axis. Brownian particles are injected at a distance *d* on both sides of the disk (dashed vertical lines) and reflected from the strip walls at *y* = ±*a*/2. (**B**) Splitting probability (normalized flux) at window 2 with angle *θ*_1_ = 0, (**C**) *θ*_1_ = *π*/2, and (**D**) *θ*_1_ = *π*.

In contrast, when the disk is located in a narrow strip (Fig. 2A), the difference of fluxes between the two windows converges asymptotically to a finite difference Δ*P* depending on the strip width *a*, even for large source distances *L* ≥ 100*R* (see Fig. 2B–D). Indeed, the fluxes hardly show any dependence on source distance *L*. The narrow funnel^16^ between the strip and the disk prevents Brownian particles to reach a window located on the opposite side of the disk, leading to the observed effects.

### Sensitivity of detection

To further investigate how the window positions could influence the recovery of the source location, we estimated the maximum distance between the source and the disk containing two absorbing windows located at position ***x***_1_, ***x***_2_ that gives a significant difference of probability flux. For that purpose, we define the sensitivity ratio as

8 $$r({{\boldsymbol{x}}}_{1},{{\boldsymbol{x}}}_{2},{{\boldsymbol{x}}}_{0})=\frac{|{P}_{1}({{\boldsymbol{x}}}_{1},{{\boldsymbol{x}}}_{2},{{\boldsymbol{x}}}_{0})-{P}_{2}({{\boldsymbol{x}}}_{1},{{\boldsymbol{x}}}_{2},{{\boldsymbol{x}}}_{0})|}{{P}_{1}({{\boldsymbol{x}}}_{1},{{\boldsymbol{x}}}_{2},{{\boldsymbol{x}}}_{0})+{P}_{2}({{\boldsymbol{x}}}_{1},{{\boldsymbol{x}}}_{2},{{\boldsymbol{x}}}_{0})}$$

(note that here *P*_1_(***x***_1_, ***x***_2_, ***x***_0_) + *P*_2_(***x***_1_, ***x***_2_, ***x***_0_) = 1). The domain of sensitivity for a threshold *T*_*h*_ is the interior of the two-dimensional region

9 $${D}_{S}=\{{{\boldsymbol{x}}}_{0}\,{\rm{such}}\,{\rm{that}}\,r({{\boldsymbol{x}}}_{1},{{\boldsymbol{x}}}_{2},{{\boldsymbol{x}}}_{0})\ge {T}_{h}\}.$$

**Figure 3:**
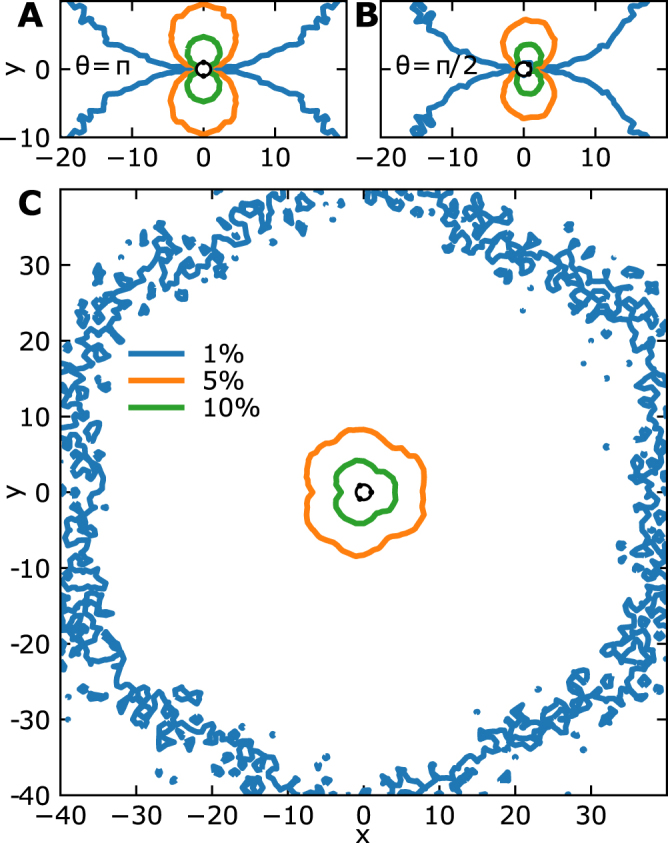
Detectable region and contours for small windows on a disk. (**A**) Two windows are placed on a disk with angular spacing *θ* = *π*. The contours indicate the position for a threshold *T*_*h*_ = 1%, 5% and 10%, given by the normalized flux difference or probability (8). (**B**) Two windows are placed with an angle *π*/2 apart. (**C**) Three windows placed 2*π*/3 apart. The contours of *D*(*S*) are given by *r*_3_ = *T*_*h*_ (relation (12)).

We plotted the boundary of the region *D*_*S*_ for two absorbing windows symmetrically positioned (Fig. 3A) and when the angle is *θ*_12_ = *π*/2 (Fig. 3B) for *T*_*h*_ = 1%, 5% and 10%. The region *D*_*S*_ consists of two connected components and no detection (for a threshold below *T*_*h*_) is possible outside *D*_*S*_. Interestingly, with a 1% precision, the domain is around 20× the size of the detecting disk. Beyond this distance, no detection is possible. The detection sensitivity for a given source location ***x***_0_ and optimal window placement is defined by

10 $$f({{\boldsymbol{x}}}_{0})=\mathop{{\rm{\max }}}\limits_{{{\boldsymbol{x}}}_{1},{{\boldsymbol{x}}}_{2}}|{P}_{1}({{\boldsymbol{x}}}_{1},{{\boldsymbol{x}}}_{2},{{\boldsymbol{x}}}_{0})-{P}_{2}({{\boldsymbol{x}}}_{1},{{\boldsymbol{x}}}_{2},{{\boldsymbol{x}}}_{0})|.$$

The maximum is achieved for a window configuration aligned with the position of the source and symmetric with respect to the center of the disk centered at the origin. An explicit computation with ***x***_2_ = −***x***_1_, |***x***_1_| = |***x***_2_| = *R* gives

11 $$f({{\boldsymbol{x}}}_{0})=\frac{2R}{|{{\boldsymbol{x}}}_{0}|\,\mathrm{log}\,\frac{2R}{\varepsilon }}+o(\frac{1}{|{{\boldsymbol{x}}}_{0}|}),$$

where *R* is the radius of the disk. Hence, the detection sensitivity decreases as the reciprocal of the distance to the source *L* = |***x***_0_|. With three windows, detection is possible if at least one of the difference between the splitting probability is higher than the threshold *T*_*h*_. We thus define the new sensitivity ratio using

12 $${r}_{3}({{\boldsymbol{x}}}_{1},{{\boldsymbol{x}}}_{2},{{\boldsymbol{x}}}_{3},{{\boldsymbol{x}}}_{0})=\frac{{\rm{\max }}\,\{|{P}_{1}-{P}_{2}|,|{P}_{1}-{P}_{3}|,|{P}_{2}-{P}_{3}|\}}{{P}_{1}+{P}_{2}+{P}_{3}},$$

with *P*_1_, *P*_2_ and *P*_3_ defined above depending on ***x***_1_, ***x***_2_, ***x***_3_ and ***x***_0_. This definition accounts for the symmetric positioning of absorbing windows located on the boundary of the disk. The region where the source is detected surrounds the detecting disk Ω. Numerical simulations show the region *D*_*S*_ defined in Eq. (9) for the function *r*_3_ with windows positioned at the corners of an equilateral triangle is now connected and seems to extend to 40 times the size of the detecting disk (Fig. 3C).

### Diffusion-triangulation of the gradient source

**Figure 4:**
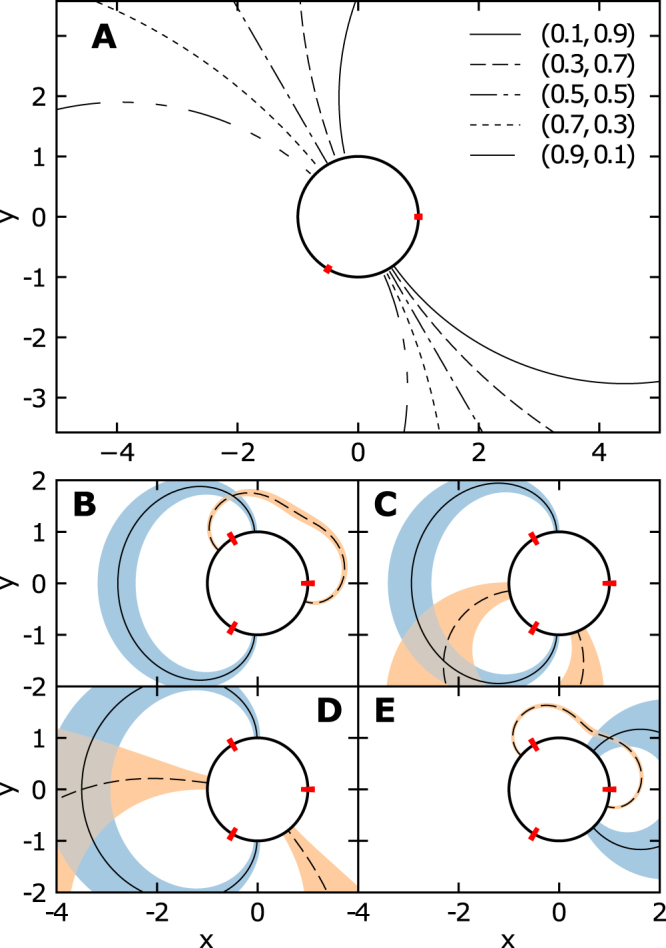
Recovery or triangulation of the source position from fluxes to small absorbing windows. (**A**) Two windows are positioned on a detecting disk with an angular spacing of 2*π*/3. This arrangement allows recovery for the position ***x***_0_ only a one dimensional curve. The curves are the ensemble of solutions for ***x***_0_ computed from equation (6) for the fluxes (*P*_1_, *P*_2_) displayed in the figure legend. (**B**–**E**) Intersection of the solid and dashed curves computed from equation (6) for three absorbing windows. In that case, a unique position is recovered for the source position ***x***_0_. We provide several examples: the original source positions are (**B**) ***x***_0_ = (−0.5, 1.7), (**C**) ***x***_0_ = (−2.3, −1.5), (**D**) ***x***_0_ = (−3.5, 0) and (**E**) ***x***_0_ = (1, 1). The amplitude of fluctuations for a fixed uncertainty *η* = 0.15 added to the fluxes (chosen arbitrarily for illustration purposes) is represented in shaded areas. The resulting uncertainty in the recovered source position corresponds to overlap of the shaded regions.

Reconstructing the location of the source in the detectable region *D*_*S*_ from the steady-state probability fluxes, requires inverting Eq. (6) and to find ***x***_0_ when the fluxes at the windows (receptors) are known. With two windows located on a detecting disk and using the expression of the Green’s function (7), we obtain a one dimensional curve (Fig. 4A). Therefore, at least three windows are required to recover the point source. We indeed found that the source is located at the intersection of two curves (Fig. 4B). Indeed, since *P*_1_ + *P*_2_ + *P*_3_ = 1, we only need to estimate *P*_1_ and *P*_2_, obtained from Eq. (3) by the same matched asymptotic method as for two windows, except that now we have to invert a three-dimensional matrix equation (see SI). We then use the expression of the Green’s function given in Eq. (7) to invert the equations and the intersection point is found numerically via MINPACK’s multidimensional nonlinear root finding method *hybrj*^21^. Interestingly, fluctuations in the probability fluxes *P*_*i*_ (implemented by changing *P*_*i*_ → [1 ± *η*]*P*_*i*_ in SI equations S1) yields large, nonlinear and spatially inhomogeneous uncertainty in the reconstructed source position as shown in the overlap of the shaded areas in Fig. 4B. Indeed, in two dimensions, reconstructing the position (i.e. two unknown coordinates) requires two intersecting curves. Hence, three windows are needed, yielding the source position as the intersection point of two curves (Fig. 4B). Interestingly, fluctuations in the probability flux lead to large, nonlinear and spatially inhomogeneous uncertainty in the recovery of the source position as shown in Fig. 4B–F.

## Discussion and Conclusion

The ability of a cell to sense a gradient concentration is mediated by the binding of Brownian molecules to receptors^11,22^. Computing the diffusion fluxes via homogenizing over local receptor positions^2,23,24^, renders a recovery of any directional information impossible, as it assigns the same flux to the entire boundary of the detecting disk. Based on Narrow Escape Theory^17–19^, we estimated the probability fluxes on each individual receptor window separately and found that in two dimensions (i.e. a flat environment), the direction of the source can be recovered. This mechanism requires a comparison between the fluxes of at least three boundary receptors. In addition, the Green’s function method allows us to estimate the boundary of the region of sensitivity characterized by a difference of fluxes between receptors being larger than a predefined sensitivity threshold. The sensitivity decays with the reciprocal of the distance to the source according to Eq. (11). Furthermore, we evaluated the effect of the external geometry on the threshold of sensitivity: although a disk of radius R with absorbing windows cannot sense the source position located beyond ten cell radii, in a narrow band, the detection is possible due to narrow passages for the diffusing molecules^16^, as long as one detecting window is facing away from the source.

The present results have several applications, such as growth-cone navigation inside the developing brain. Neurons have to travel millimeters to centimeters to find the correct cortical regions and to form synaptic connections^2,3,25^. We propose that narrow extracellular tubes formed by neurons and glial cells probably allow the genesis of shallow gradients detected by receptors located on the growth cone, although it is not known precisely how these receptors are organized. The dynamics of the growth cone including moving protrusions is certainly an additional mechanism worthy of further investigation in context of direction sensing, but should dominate for short-range distances only. Finally, we suggest here that growth-cone gradient assays in restricted environments could reveal how narrow passages, generated between neurons modulate the search for a final destination. These passages could explain the amplification of the gradient sensing much further than the 10 cell radii, as predicted by the diffusion hypothesis that we have explored here.

Although we focussed on two and three windows only, the results would be similar for two or three receptor clusters^17^. The model we used here was developed in the fast binding limit without rebinding^4^. It remains a challenge to apply the present theory to understand how sperm^5^ or neurite growth^25^ localize the source of their cues. We limited the present approach to the initial level of source detection, however the asymmetry of receptor detection needs to persist in the downstream transduction, which certainly is another key question to investigate. Future works should also include the time scales of binding and unbinding events to receptors and explore the consequence of dimension 3, that require generalizing the Narrow escape approach^14,17^.

## Electronic supplementary material

Supplementary Information
