# Supplementary material for: Reconstructing the gradient source position from steady-state fluxes to small receptors
Source: Sci Rep. 2018 Jan 17;8:941. doi: 10.1038/s41598-018-19355-5 (PMC5772644; doi:10.1038/s41598-018-19355-5)
Supplement: Supplementary file 1 — Supplementary Information [file 41598_2018_19355_MOESM1_ESM.pdf]

Reconstructing the gradient source position from steady-state fluxes to small receptors

U. Dobramysl[1], D. Holcman[2] *

November 28, 2017

Supplementary material: Recovery of the source position for $N = 3$ windows

The distance between two window positions $\boldsymbol{x}_i$ and $\boldsymbol{x}_j$ is given by

$$d_{ij} = |\boldsymbol{x}_i - \boldsymbol{x}_j|.$$

We then define the scaled quantities

$$h_{ij} = \log \frac{d_{ij}}{\varepsilon},$$

the product vector

$$F = -(h_{23}[h_{12} + h_{13} - h_{23}], h_{13}[h_{12} - h_{13} + h_{23}], h_{12}[-h_{12} + h_{13} + h_{23}])^T,$$

the determinant

$$\Delta = (h_{12} + h_{13} + h_{23})^2 - 4(h_{12}h_{13} + h_{12}h_{23} + h_{13}h_{23})$$

and the matrix

$$[b] = \begin{pmatrix} -2h_{23} & -h_{12} + h_{13} + h_{23} & h_{12} - h_{13} + h_{23} \\ -h_{12} + h_{13} + h_{23} & -2h_{13} & h_{12} + h_{13} - h_{23} \\ h_{12} - h_{13} + h_{23} & h_{12} + h_{13} - h_{23} & -2h_{12} \end{pmatrix}.$$

This gives the solution to Eq. (1) as

$$\boldsymbol{P} = \frac{1}{\Delta}(\boldsymbol{F} - \pi \boldsymbol{G}^T[b]), \tag{S1}$$

*[1] Cancer Research UK Gurdon Institute, University of Cambridge, United Kingdom [2] Ecole Normale Supérieure, 46 rue d'Ulm 75005 Paris, France and Mathematical Institute, University of Oxford, Woodstock Rd, Oxford OX2 6GG, United Kingdom.

where $\boldsymbol{P} = (P_1, P_2, P_3)^T$ and

$$\boldsymbol{G} = \begin{pmatrix} G(\boldsymbol{x}_1, \boldsymbol{x}_0) \\ G(\boldsymbol{x}_2, \boldsymbol{x}_0) \\ G(\boldsymbol{x}_3, \boldsymbol{x}_0) \end{pmatrix} .$$

We then use Eq. (7) for the expression of the Green's function, which yields three intersecting curves, one of which contains redundant information due to $P_1 + P_2 + P_3 = 1$. We numerically invert the equations and find the intersection point $\boldsymbol{x}_0$ via the multidimensional nonlinear root finding algorithm *hybrj* contained in MINPACK [21].
